# Health literacy and libraries

**DOI:** 10.29173/jchla29874

**Published:** 2025-08-01

**Authors:** Kallen Rutledge

**Affiliations:** Librarian Educator, Nova Scotia Health, Dartmouth, NS, Canada

Vardell E, Charbonneau DH, editors. **Health literacy and libraries**. Lanham, MD: Rowman & Littlefield; 2024. Hardcover: 339 p. ISBN: 978-1-5381-8078-5. Price: USD$145. Available from: https://www.bloomsbury.com/us/health-literacy-and-libraries-9781538180785/.

In a time when we are frequently bombarded with health information in a variety of formats and channels, the skills needed to manage one’s health seem to be continuously changing. In the years following the COVID-19 pandemic, librarians and information professionals across the globe continue to work towards identifying how to best support their patrons’ health literacy needs. In their well-organized and easy to read book, *Health literacy and libraries*, editors Emily Vardell and Deborah H. Charbonneau present a comprehensive compendium of essays each highlighting the importance of libraries, regardless of library type, in addressing health literacy on behalf of the populations they serve [1].

Outlining pragmatic viewpoints, each chapter in *Health literacy and libraries* provides insight into one of the many facets of the ever expanding field of health literacy. Separate chapters explore the challenges that many minorities living in the United States face, including health disparities, language barriers, and access issues. The health literacy challenges faced by young adults, caregivers, and seniors are also discussed with real-life examples of how to support their health literacy. The roles of different types of libraries and the audiences they serve, including medical students and healthcare providers, are discussed in detail, tying together themes of advocacy and collaboration to overcome common barriers of misinformation, low literacy rates, and access challenges. Chapter after chapter, the authors outline not only the “why” of prioritizing health literacy, but also offer detailed strategies on how library teams can accommodate and improve differing levels of health literacy through their everyday work and intentional programming.

Similar topics of misinformation, consumer health literacy, and advocacy are addressed in Dalrymple and Galvin’s (2020) publication, *Growing community health literacy through libraries: sharing global perspectives* [2]. However, comparatively, Vardell and Charbonneau present a more focused collection of mainly U.S. based examples, which exhibit relatable similarities to many aspects seen throughout the Canadian information landscape. Moreover, *Health literacy in libraries* also provides more in-depth coverage than the limited consumer health lens presented in St. Jean, Jindal, Liao, and Jaeger’s (2021) publication, *Roles and responsibilities of libraries in increasing consumer health literacy and reducing health disparities* [3], making it a preferred choice on any health science librarian’s reading list.

An associate professor in the School of Library and Information Management at Emporia State University in Kansas, co-editor Emily Vardell co-authored two chapters in *Health literacy and libraries*, including chapter two on how health literacy can be measured, and chapter three on health insurance literacy. She holds a PhD in Information Science from the University of North Carolina at Chapel Hill, and a Master of Library Science from Texas Woman’s University. Alongside Vardell as co-editor, Deborah H. Charbonneau is well-cited in the growing research field of health literacy. Charbonneau is a professor and chair of the Master of Library and Information Science program at Wayne State University in Michigan, where she also earned her PhD in sociology. Charbonneau also co-authored the final chapter, “Partnerships for health literacy: information access, training, and programming” (chapter 23).

In a short preface identifying their goal to provide a tangible and relevant guide to understanding health literacy, Vardell and Charbonneau outline how the book is organized. Their collection includes 23 chapters authored by a wide range of information professionals, scientists, and other specialists. The chapters are divided into five main sections, each starting with summarized key points and concluding with insightful discussion questions that often encourage the reader to carefully reflect on their own experiences as they relate to the text.

*Health literacy and libraries* starts out by outlining the fundamentals needed to adequately prepare the reader for more in-depth discussions to follow. Chapter one introduces the history of the term “health literacy” in the United States, and chapter two outlines common health literacy measurement tools. Section two of the book sheds light on often overlooked but highly relevant sub-types of health literacy: health insurance literacy (chapter three) and mental health literacy (chapter four). Section two, chapter five focuses on health literacy and health misinformation. These concepts are described by chapter authors Haberstroh and Campbell Rice as having a “multidimensional relationship” [4].

Section three of the book is made up of nine chapters shedding light on different affected communities, each with its own unique experience but one common denominator with significant impact on health outcomes: low health literacy. Chapter six outlines how low health literacy has affected LGBTQ+ communities in North America, followed by chapter seven focusing on Latinx and Hispanic communities, which reminds the reader to avoid forming common misconceptions on literacy based solely on race or culture. Chapter eight focuses on the experiences shared by many Black communities in the U.S., and chapters 10 and 11 focus on those of immigrants, refugees, and migrants. Chapter nine explores the experience of public libraries in China on providing health literacy education. Chapters 12, 13, and 14 examine the health literacy experience of patrons at various life stages, identifying that older adults, young adults, and caregivers have different informational needs and as such, may require different strategies to engage and support them.

Section four, made up of chapters 15, 16, and 17, examines the differing experiences of academic, public, and hospital libraries in promoting health literacy. Chapter authors describe how information professionals working in each of these sectors can best assist their patrons to improve their health literacy skills. From how to equip future healthcare providers, to conducting a community needs assessment, chapter authors provide readers with the tools needed to foster health literacy in their respective environments.

The final section of *Health literacy and libraries*, “Health literacy in practice,” highlights a variety of approaches to help address health literacy needs in a wide array of contexts. Examples of real-world initiatives found in the final chapters were of the most interest to me since my day-to-day focus is less on improving the health literacy of individual patrons than it is on improving health literacy awareness in the organizational context and how to best support registered health professionals. Reoccurring themes emerge throughout chapters 18 to 23, highlighting the role librarians hold as agents of change and advocacy. From intentional book clubs that choose selections with health topics weaved into their stories, to educating medical students on how to dispel misinformation when it rears its social media fueled head, the final chapters outline a variety of practical strategies any information professional could adapt to suit their own organizational context.

While chapter three is less relevant within the Canadian context (where we have free universal healthcare) the remaining chapters in this book hold significant relevance to health science librarians in North America, including myself. *Health literacy in libraries* provides readers with a clear understanding of the many barriers and facilitators to improving health literacy and would be a valuable resource for information professionals who are new to the field or who are experienced with health literacy pedagogy. Vardell and Charbonneau have selected works that provide practical examples that are relevant in a post-global pandemic world, providing the needed insight to create an actionable health literacy strategy.
